# Rapid Increase in Carriage Rates of *Enterobacteriaceae* Producing Extended-Spectrum β-Lactamases in Healthy Preschool Children, Sweden

**DOI:** 10.3201/eid2410.171842

**Published:** 2018-10

**Authors:** Johan Kaarme, Hilde Riedel, Wesley Schaal, Hong Yin, Tryggve Nevéus, Åsa Melhus

**Affiliations:** Uppsala University, Uppsala (J. Kaarme, H. Riedel, W. Schaal, T. Nevéus, Å. Melhus);; Falu Hospital, Falun, Sweden (H. Yin)

**Keywords:** carriage rates, Enterobacteriaceae, bacteria, extended-spectrum β-lactamases, ESBLs, healthy preschool children, AmpC, preschool children, cephalosporin resistance, antimicrobial resistance, whole-genome sequencing, enteric infections, respiratory infections, Sweden

## Abstract

By collecting and analyzing diapers, we identified a >6-fold increase in carriage of extended-spectrum β-lactamase (ESBL)–producing *Enterobacteriaceae* for healthy preschool children in Sweden (p<0.0001). For 6 of the 50 participating preschools, the carriage rate was >40%. We analyzed samples from 334 children and found 56 containing >1 ESBL producer. The prevalence in the study population increased from 2.6% in 2010 to 16.8% in 2016 (p<0.0001), and for 6 of the 50 participating preschools, the carriage rate was >40%. Furthermore, 58% of the ESBL producers were multidrug resistant, and transmission of ESBL-producing and non–ESBL-producing strains was observed at several of the preschools. Toddlers appear to be major carriers of ESBL producers in Sweden.

The rapid dispersion of multidrug-resistant bacteria is considered one of the main threats to global public health (*1*), and it shows no sign of abating. Members of the family *Enterobacteriaceae* harboring extended-spectrum β-lactamases (ESBLs) are playing a major role in this development. These bacteria have spread quickly worldwide as a result of their mobile genetic elements or clonal dissemination (*2*), and the resulting infections have become a clinical challenge. Consequently, illnesses and deaths caused by these bacteria and related healthcare costs have increased (*3*,*4*).

Transferable enzymes of the ESBL type have been reported since the early 1980s (*5*). These enzymes confer resistance to penicillins, cephalosporins, and monobactams, but not to cephamycins or carbapenems, and are inhibited by β-lactam inhibitors (*6*). Their prevalence is steadily increasing. During the past 2 decades, CTX-M type enzymes have become the most predominant, followed by the previously dominating SHV and TEM types (*2*). The dissemination of ESBL producers of the CTX-M type is problematic because co-resistance to other major classes of antimicrobial drugs is frequent (*7*). The European Centre for Disease Prevention and Control has declared that the increasing prevalence of ESBLs in invasive isolates in Europe is particularly worrisome (*8*).

Most studies on ESBL producers have focused on hospitalized and adult patients, some have explored the prevalence for healthy persons, and even fewer included children (*8**–**10*). In 2010, we conducted a study on fecal carriage of ESBL-producing *Enterobacteriaceae* for healthy preschool children in Sweden and reported a prevalence of 2.6% (*11*). Recent studies conducted in other countries have indicated diversified but sometimes high prevalence and spread of ESBL producers, in which daycare centers have been suggested to constitute possible reservoirs (*12*,*13*).

The primary purpose of this follow-up study was to investigate whether the prevalence of ESBL producers had increased in our community-based pediatric population during the past 6 years. In addition, we explored whether our previous indications of transmission of ESBL producers between children in preschools could be confirmed.

## Materials and Methods

### Settings and Study Design

During August 2016, we conducted a prospective follow-up study in Uppsala, Sweden. All 71 municipal preschools in the central parts of the city were invited to participate. In 2015, a total of 80% of all children 1–5 years of age in Uppsala County attended preschool. Of these children, 74% were enrolled in municipal preschools (mean 16.7 children in each group). The remaining children attended private preschools or other child care providers or stayed at home (*14*). Most attendees spent 6–8 hour/workday in the preschools.

Before the study was conducted, an advisory statement was obtained from the Regional Ethics Committee that established diapers as biologic waste. Therefore, no ethics consent was needed. Information about the study was thereafter sent to the directors of the participating preschools for further distribution to the staff and parents. In preschools in which staff and parents gave their verbal consent, 1 diaper/child was collected the morning after defecation. The time span from defecation to processing of the samples was 16–25 hours. Each diaper was marked with the age of the child to avoid duplicates. Approximately 1 month later, we revisited the preschools and collected environmental samples from water traps of washbasins, toilet flush buttons, and lunch tables.

Statistics on antimicrobial drug consumption in Uppsala County were provided by the Swedish Strategic Programme for the Rational Use of Antimicrobial Agents and Surveillance of Resistance (https://isim.ku.dk/staff/vip/?pure=en%2Fpublications%2Fstrama-the-swedish-strategic-programme-for-the-rational-use-of-antimicrobial-agents-and-surveillance-of-resistance(95ae88b0-93b5-11dd-86a6-000ea68e967b).html). Information for prescriptions of antimicrobial drugs used in Uppsala County and drug requisitions to Uppsala University Hospital in 2015 was also provided by this program.

### Bacteria and Media

After collection, diapers were transported to the Department of Clinical Microbiology at Uppsala University Hospital. Stool was streaked onto MacConkey agar plates (Acumedia, Lansing, MI, USA) containing a cefotaxime disc (5 μg; Oxoid Ltd., Basingstoke, UK). Stool was also inoculated into 2 tubes containing Luria–Bertani broth (Becton, Dickinson and Co., Sparks, MD, USA) supplemented with cefpodoxime (5 μg/mL) or ertapenem (1.25 μg/mL) (Oxoid Ltd.). After incubation at 37°C for 24 hours, we semiquantified growth in the inhibition zones on MacConkey agar plates as follows: light, 1–10 CFU; moderate, 11–100 CFU; and heavy, >100 CFU. The broth was plated onto cysteine–lactose electrolyte-deficient agar plates (Becton, Dickinson and Co.) that contained cefotaxime (5 μg) and ceftazidime (10 μg) discs for selection of ESBL producers and imipenem (10 μg) and ceftazidime (10 μg) discs for selection of carbapenemase producers. We identified colonies growing in the inhibition zones after 24 hours of incubation to the species level by using matrix-assisted laser desorption/time-of-flight mass spectrometry.

Environmental samples were collected with cotton swabs and inoculated on site into Luria–Bertani broth. Subsequent handling was identical with that for fecal isolates.

### Antimicrobial Drug Susceptibility Testing

We determined antimicrobial drug susceptibility for all isolates that showed growth in the inhibition zones of cefotaxime, ceftazidime, or imipenem by using the disc diffusion method and breakpoints recommended by the European Committee on Antimicrobial Susceptibility Testing (*15*). We tested mecillinam, amoxicillin/clavulanic acid, piperacillin/tazobactam, cefoxitin, cefotaxime, ceftazidime, cefepime, aztreonam, meropenem, gentamicin, tobramycin, amikacin, nalidixic acid, ciprofloxacin, trimethoprim, cotrimoxazole, nitrofurantoin, and tigecycline. We defined multidrug resistance as resistance to >2 antimicrobial drug classes, in addition to resistance to cephalosporins, penicillins, and monobactams.

We performed phenotypic confirmation of ESBL production as described (*16*). Isolates resistant to cephalosporins but not inhibited by clavulanic acid or cefoxitin were tested by using a multiplex PCR described by Pérez-Pérez and Hanson to detect plasmid-mediated AmpC (*17*).

### Repetitive Element Palindromic PCR

To explore genetic relatedness between *Escherichia coli* isolates from the same preschool, we performed a previously described repetitive element palindromic PCR (rep-PCR) (*11*) with a slight modification: only primer ERIC2 (5′-AAGTAAGTGACTGGGGTGAGCG-3′) was used. We analyzed 86 isolates from 18 preschools with the largest number of diapers with susceptible *E. coli*. A total of 45 isolates from 13 preschools were ESBL producers; the remaining 41 isolates were fully susceptible to cephalosporins and meropenem. We collected these susceptible isolates from 4 preschools that had no ESBL-positive isolates (n = 6, n = 12, n = 6, and n = 10) and 1 preschool with 1 ESBL-positive isolate (n = 7). These isolates were included as controls to determine whether transmission occurred between children independent of ESBL production.

### Whole-Genome Sequencing

To determine ESBL types and genetic relatedness, we subjected 43 ESBL-producing *E. coli* isolates from 25 preschools to whole-genome sequencing. Isolates representative of each DNA pattern determined by the rep-PCR were chosen for each preschool. For confirmation of the performance of the rep-PCR, all ESBL producers from 2 preschools (n = 3 and n = 4) and all ESBL producers from 2 children (n = 2 and n = 2) were also completely sequenced.

We prepared DNA by using the MagAttract DNA mini M48 Kit (QIAGEN, Solna, Sweden) and the BioRobot M48 (GenoVision, West Chester, PA, USA). We sequenced genomes of selected isolates by using IonTorrent (Life Technologies, Carlsbad, CA, USA) and a read length of 400 bp, according to the manufacturer’s instructions. We assembled reads into a draft genome using the AssemblerSPAdes Plugin in TorrentSuite Version 4.2 (Thermo Fisher Scientific, Waltham, MA, USA) and the recommended settings of Life Technologies. We constructed databases for each of the assembled genomes (*18*). Sequence data were edited by using the Emboss suite (*19*) and the *E. coli* Multilocus Sequence Typing website (*20*).

We aligned whole-genome sequences with a common reference sequence (GenBank accession no. NC_010473.1) by using ABACAS (*21*) with gaps omitted. These sequences were separated by strain type according to multilocus sequence typing. Within each strain type, differences between samples were further refined by counting the number of single-nucleotide polymorphisms (SNPs) between each pair. SNP determination was performed by using MUMmer (*22*).

### Statistical Analysis

We used the 2-tailed Fisher exact test to compare groups. Differences were considered significant if p<0.05.

## Results

### Study Population

A total of 58 (82%) of 71 municipal preschools agreed to participate in the study; the preschools were evenly distributed across the city. Four of the preschools were excluded because of no delivery of diapers on the day of collection, and 4 others were excluded because of a sudden relocation of children after a fire, making it impossible to follow the epidemiology. Thus, of the 71 invited municipal preschools, 50 (70%) delivered samples that were included in the study.

**Figure 1:**
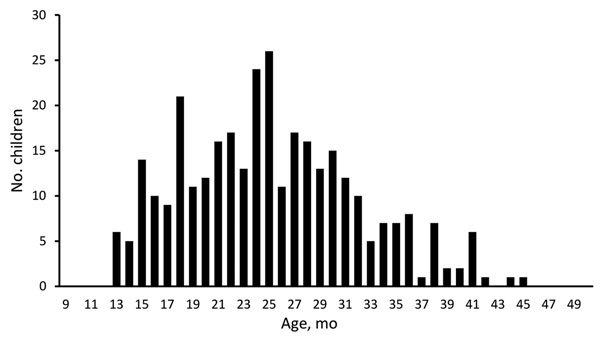
Age distribution of healthy preschool children in study of rapid increase in carriage rates of *Enterobacteriaceae* producing extended-spectrum β-lactamases, Sweden.

The number of diapers collected from each preschool ranged from 1 to 18 (median 6 diapers/preschool). The collection yielded 334 stool samples and 204 environmental samples. The age range of participating children was 13–45 months (median 25 months) (Figure 1); 8 samples had no information about the age of the child.

### Cefotaxime-Resistant Isolates

**Figure 2:**
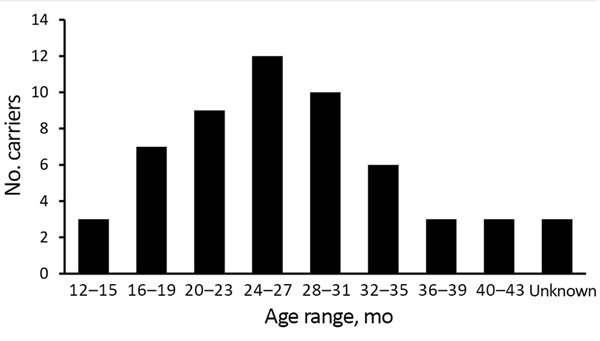
Age distribution of extended-spectrum β-lactamase carriers in study of rapid increase in carriage rates of *Enterobacteriaceae* producing extended-spectrum β-lactamases, Sweden.

Of 334 children, we found 67 (20.1%) from 32 preschools who had cefotaxim-resistant *Enterobacteriaceae* isolates in their stool. Of these 67 children, 56 (16.8%) had >1 ESBL producer (Figure 2) and 15 (4.5%) had >1 AmpC producer. We isolated 62 ESBL-producing and 15 AmpC-producing *Enterobacteriaceae* strains.

The number of carriers of ESBL-producing isolates from a single preschool ranged from 1 to 7, yielding a carriage rate of 0%–80% for the included preschools. For 6 of the preschools, the carriage rate was >40%, and in 18 preschools, none of the children were ESBL carriers. The median detection rate for the preschools was 13% positive samples.

Most (n = 47) of the ESBL-positive children had 1 ESBL producer, but 5 children had 2 ESBL-producing strains at the same time, either 2 *E. coli* strains (n = 3) or a combination of *E. coli* and *Klebsiella pneumoniae* (n = 2). For 4 children, both ESBL- and AmpC-type enzymes were detected. One child had 2 ESBL producers (*E. coli* and *K. pneumoniae*) and 1 AmpC producer (CITM-positive *E. coli*).

Eleven children had only an AmpC-producing isolate. Thus, the total number of detected cefotaxime-resistant AmpC-producing *Enterobacteriaceae* isolates was 15 (4.5%). These isolates had the following species distribution: *E. coli* (n = 7), *Enterobacter cloacae* (n = 6), *Citrobacter freundii* (n = 1), and *Citrobacter brakii* (n = 1). For the *E. coli* isolates, plasmid-mediated resistance predominated; 4 isolates were DHAM positive and 1 isolate was CITM positive.

Thirty (69.8%) of the 43 fully sequenced ESBL-producing *E. coli* isolates harbored CTX-M-15, five had CTX-M-14 or CTX-M-14–like β-lactamases, four had CTX-M-55, three had CTX-M-57, and one had CTX-M-27. Eleven of the isolates harbored concomitant TEM enzymes: four had TEM-2, two had TEM-55, two had TEM-56, two had TEM-109, and one had TEM-4.

The 3 most common multilocus sequence types (STs) for the ESBL producers for which whole-genome sequencing was performed were ST131 (n = 11, 25.6%), followed by ST10 and ST354 (both n = 5, 11.6%). ST131 was present in 9 preschools, ST10 in 4, and ST354 in 3. Other STs represented were ST38 or the clonal complex containing ST58, ST127, ST155, ST328, ST349, ST362, ST450, ST602, ST998, ST1423, ST1634, ST2064, ST3877, and ST6448.

The 204 environmental samples yielded only 1 AmpC-producing *E. coli* isolate from a washbasin in 1 of the diaper-changing rooms of 1 preschool. This preschool had 3 fecal samples with ESBL/AmpC production, which was unrelated to the AmpC-producing *E. coli*.

### Transmission of *E. coli* Isolates

We detected identical strains in both preschools with and without ESBL producers. If there were 2–3 ESBL-positive children in a preschool, they, as a rule, had the same strain. With increasing numbers of ESBL-positive children in a school, the likelihood increased for >1 strain being involved. In the preschool that had the highest total number of ESBL-positive children (n = 7), 4 ESBL strains were identified, of which 3 were *E. coli*. All *E. coli* strains had been transmitted to 1 or 2 additional children, but the ESBL-positive *K. pneumoniae* strain was detected in only 1 child. We found no correlation between the quantity of growth in the inhibition zone of cefotaxime and the tendency to spread.

The 3 most common STs were further analyzed to rule out transmission between preschools. ST10 isolates were unambiguously different (SNPs 1,200–15,000). ST38 isolates were generally more closely related than were ST10 isolates but were insufficient to support direct transmission. The closest pair of STs showed a difference of ≈300 SNPs. The most frequent ST group, ST131, held several moderately to closely related isolates. Three isolates formed the closest group (SNPs <45). Another group of 3 isolates was moderately related to the other 3 samples and to each other (SNPs ≈100–350). The remaining isolates formed a separate group with SNPs >400.

In preschools that had susceptible *E. coli* strains, <2 children had the same strain, and <3 strains were present in the same children. Only 1 preschool showed no spread of *E. coli* strains between the children. In this preschool, there were no children who had ESBL producers.

### Antimicrobial Drug Resistance

**Figure 3:**
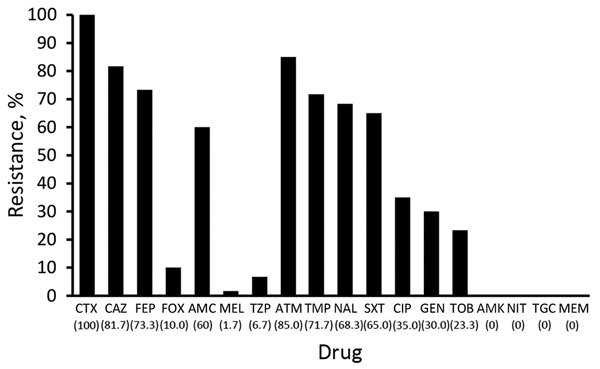
Antimicrobial drug resistance for extended-spectrum β-lactamase–producing *Enterobacteriaceae* isolates from 62 healthy preschool children, Sweden. Values in parentheses along the x-axis are percentages. AMC, amoxicillin/clavulanic acid; AMK, amikacin; ATM, aztreonam; CAZ, ceftazidime; CIP, ciprofloxacin, CTX, cefotaxime; FEP, cefepime; FOX, cefoxitin; GEN, gentamicin, MEL, mecillinam; MEM, meropenem; NAL, nalidixic acid; NIT, nitrofurantoin; SXT, sulfamethoxazole/trimethoprim; TGC, tigecycline; TOB, tobramycin; TZP, piperacillin/tazobactam; TMP, trimethoprim.

**Figure 4:**
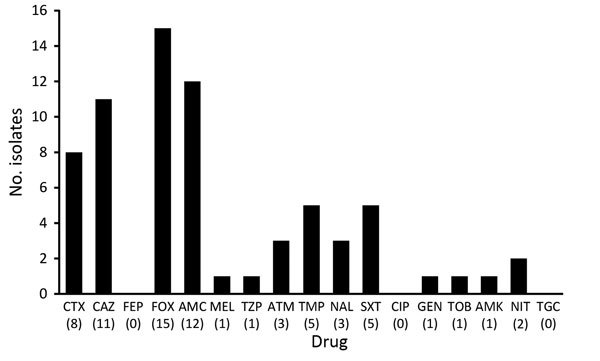
Antimicrobial drug resistance for AmpC-producing *Enterobacteriaceae* isolates from 15 healthy preschool children, Sweden. Values in parentheses along the x-axis are percentages. AMC, amoxicillin/clavulanic acid; AMK, amikacin; ATM, aztreonam; CAZ, ceftazidime; CIP, ciprofloxacin, CTX, cefotaxime; FEP, cefepime; FOX, cefoxitin; GEN, gentamicin, MEL, mecillinam; NAL, nalidixic acid; NIT, nitrofurantoin; SXT, sulfamethoxazole/trimethoprim; TGC, tigecycline; TOB, tobramycin; TZP, piperacillin/tazobactam; TMP, trimethoprim.

Thirty-six (58%) of the 62 ESBL-producing isolates and 3 (20%) of the 15 AmpC-producing isolates were multidrug resistant. For ESBL producers, the most frequent drug resistance was resistance to trimethoprim (71.7%) or cotrimoxazole (65.0%), followed by the quinolones (nalidixic acid 68.3% and ciprofloxacin 35%) (Figure 3). For AmpC-positive isolates, the pattern was similar but the overall resistance rates were lower, with the exception of nitrofurantoin (Figure 4).

### Consumption of Antimicrobial Drugs

Uppsala County had the second highest number of prescriptions and sales on hospital requisitions in Sweden in 2015: a total of 13.9 defined daily doses/1,000 person-days. Of these doses, 4.87 defined daily doses/1,000 person-days were prescribed to children <1–17 years of age and 5.58 defined daily doses/1,000 person-days to children 1–5 years of age. Most (93.5%) of the antimicrobial drugs were prescribed for outpatients. The most commonly used drug by children was penicillin V, followed by amoxicillin with or without clavulanic acid and flucloxacillin (Table).

**Table:** **Table.** Antimicrobial drug use for children <1–17 years of age, Uppsala County, Sweden, 2015*

Drug	DDD/1,000 person-days
Phenoxymethylpenicillin	2.44
Amoxicillin	0.59
Flucloxacillin	0.56
Amoxicillin/clavulanic acid	0.20
Trimethoprim/sulfamethoxazole	0.18
Cefadroxil	0.16
Erythromycin	0.13
Pivmecillinam	0.08
Clindamycin	0.06
Ceftibuten	0.06
Nitrofurantoin	0.05
Azithromycin	0.05
Ciprofloxacin	0.05
Cloxacillin	0.03
Meropenem	0.02
Cefotaxime	0.02
Metronidazole	0.02
Benzylpenicillin	0.01
Vancomycin	0.01
Piperacillin/tazobactam	0.01

*The following drugs had DDD values <0.01/1,000 person-days: coxycycline, ampicillin, cefuroxime, ceftazidime, ceftriaxone, ertapenem, imipenem, ceftaroline fosamil, tobramycin, gentamicin, moxifloxacin, colistin, daptomycin, rifampin, retapamulin, and fusidic acid. DDD, defined daily dose.

## Discussion

We investigated the carriage rate of ESBL producers in healthy preschool children and whether preschools might serve as a reservoir for ESBL producers in Sweden. We analyzed 334 stool samples, and results showed that the prevalence of ESBL-producing *Enterobacteriaceae* was 16.8% in the study population, which was a >6-fold increase compared with findings of the study we conducted in 2010 in the same setting and with identical handling of samples (*11*). Such a rapid increase of ESBL producers in a country with a low level of endemicity, and within a relatively infection-prone group with few treatment options, is worrisome, especially because 58% of the isolates were multidrug resistant. A similar increase, but on a lower level (from 4.8% to 10.2%), has been reported for healthy children in France during 2010–2015 (*23*).

Studies on fecal carriage of cefotaxime-resistant *Enterobacteriaceae* for healthy adults have shown widely divergent results, from a few percent up to >90% carriage, and with a pronounced geographic variation (*24*). A recent study in Sweden on elderly persons living in their own homes showed an ESBL prevalence of 8.7% (*25*), which was almost half the prevalence we found in our study.

There are few comparable studies on carriage rates of ESBL producers for healthy children. Pallecchi et al. reported an increase from 0.1% to 1.7% for children in Bolivia and Peru during 2002–2005 (*26*), and a study in Portugal reported a prevalence of 2.7% for healthy children during 2007 (*27*). Subsequent studies from the Netherlands, France, Spain, Laos, Lebanon, and Germany have reported carriage rates of 4.6%–49.6%; the highest prevalences were 24.8% for preschool children in Laos and 49.6% for healthy infants in Lebanon (*12*,*23*,*28*–*32*).

The prevalence of ESBL producers varied between preschools in this study (0%–80%, median 13% positive samples). Preschools with high prevalences were found in all parts of Uppsala, Sweden, as was transmission of ESBL producers in children. However, transmission was not exclusive for ESBL producers. Cephalosporin-susceptible *E. coli* strains were transmitted in all but 1 of the investigated preschools, which indicated that prerequisites for spread of ESBL producers were present.

Attendance at preschools and daycare centers has been shown to increase the risk for transmission of microbes and gastrointestinal infections (*33*,*34*). Furthermore, a recent study in the Netherlands reported an increased risk for colonization with ESBL producers in household members if the children attend daycare centers (*35*). However, Birgy et al. reported an inverse relationship (*23*). All participating preschools had uniformly high standards of hygiene, although we did not study compliance with hygiene guidelines. Close contacts, crowding, difficulty in complying with basic hygiene routines, and a high likelihood for exposure to antimicrobial drugs, especially third-generation cephalosporins, for young children (for whom prescriptions in Uppsala are comparably high in Sweden, but still low in comparison with international standards) make it plausible that preschools could act as reservoirs or even hotspots for ESBL producers. To our knowledge, only 3 studies have addressed this hypothesis, but results were somewhat contradictory (*11*,*23*,*30*). No larger changes in antimicrobial drug use were detected in the population included in this study during 2010–2016. In addition, we believe that it is less likely that selective pressure is the explanation for the higher prevalence.

With the exception of 1 AmpC-producing *E. coli* that had no close genetic relatedness with isolates from the children, no other cephalosporin-resistant isolate was identified in the environment. Therefore, it is more likely that the transmission route was person-to-person, rather than spread from environmental sources. Nevertheless, none of the strains was ESBL producing or transmitted among >3 children in a preschool. The most common ESBL-producing *E. coli* strain in the study was ST131 (*36*). Like all more frequent STs that were present in >1 preschool, the most SNPs were in the hundreds or thousands. Therefore, a common source of these strains does not appear likely, except for the 3 ST131 isolates.

Studies have confirmed a relationship between ESBL carriage and foreign travel (especially to Southeast Asia), geographic location, recent use of antimicrobial drugs, close contact with ESBL carriers, hospital care, or residency in nursing homes (*2*,*25*). Once colonized, presence of ESBL producers can have a long duration. Barreto Miranda et al. reported that 25% of travelers from Germany still carried ESBL producers after 6 months (*37*). In addition, carriage for as long as 5 years appears to be possible (*38*). However, it is not yet known how long carriage can persist in children or if it differs from carriage in adults.

The fact that our samples were collected anonymously is a major limitation because we could not evaluate risk factors such as antimicrobial drugs, hospital care, international travel, siblings, and family conditions. However, because our objectives were to explore the overall prevalence and possible transmission between attendees, underlying causes for the increase were beyond the scope of the study, but investigating these causes would be of interest in a future study. The fact that fecal and environmental samples were collected 1 month after collection of diapers is also a limitation. However, detection of ESBL-positive children, no change in routines, and a low isolation frequency for multidrug resistance makes environmental spread less likely.

In conclusion, the prevalence of ESBL-producing *Enterobacteriaceae* was explored for preschool children in Sweden. Results showed that 16.8% of the children carried ESBL producers, which was the highest prevalence ever recorded in Sweden. Toddlers appear to be major carriers for ESBL producers, and preschools might act as hotspots for these bacteria.
